# Importance of Tissue Preparation Methods in FTIR Micro-Spectroscopical Analysis of Biological Tissues: ‘Traps for New Users’

**DOI:** 10.1371/journal.pone.0116491

**Published:** 2015-02-24

**Authors:** Vladislava Zohdi, Donna R. Whelan, Bayden R. Wood, James T. Pearson, Keith R. Bambery, M. Jane Black

**Affiliations:** 1 Department of Anatomy & Developmental Biology, Monash University, Clayton, Victoria 3800, Australia; 2 Centre for Biospectroscopy and School of Chemistry, Monash University, Clayton, Victoria 3800, Australia; 3 Department of Physiology, Monash University, Clayton, Victoria 3800, Australia; University of Quebect at Trois-Rivieres, CANADA

## Abstract

Fourier Transform Infrared (FTIR) micro-spectroscopy is an emerging technique for the biochemical analysis of tissues and cellular materials. It provides objective information on the holistic biochemistry of a cell or tissue sample and has been applied in many areas of medical research. However, it has become apparent that how the tissue is handled prior to FTIR micro-spectroscopic imaging requires special consideration, particularly with regards to methods for preservation of the samples. We have performed FTIR micro-spectroscopy on rodent heart and liver tissue sections (two spectroscopically very different biological tissues) that were prepared by desiccation drying, ethanol substitution and formalin fixation and have compared the resulting spectra with that of fully hydrated freshly excised tissues. We have systematically examined the spectra for any biochemical changes to the native state of the tissue caused by the three methods of preparation and have detected changes in infrared (IR) absorption band intensities and peak positions. In particular, the position and profile of the amide I, key in assigning protein secondary structure, changes depending on preparation method and the lipid absorptions lose intensity drastically when these tissues are hydrated with ethanol. Indeed, we demonstrate that preserving samples through desiccation drying, ethanol substitution or formalin fixation significantly alters the biochemical information detected using spectroscopic methods when compared to spectra of fresh hydrated tissue. It is therefore imperative to consider tissue preparative effects when preparing, measuring, and analyzing samples using FTIR spectroscopy.

## Introduction

Fourier transform infrared (FTIR) spectroscopy and imaging is an emerging technique in the field of *ex vivo* diagnostics. In the last twenty years, the infrared spectra of single cells and intact tissues originating from dozens of species and cell types have been analyzed by several groups worldwide [1–6]. These studies have not only provided important information regarding the macromolecular contents and their distribution in a cell or tissue sample but have also demonstrated the ability of FTIR spectroscopy to differentiate between diseased and non-diseased states [7–10],determine cell cycle stage [11] and monitor cell death [12]. By measuring the absorption of infrared light by a sample, the characteristic energies and intensities of absorbance bands of cellular macromolecules can be detected and assigned, including carbohydrates [13–15] lipids [16], proteoglycans [17,18], collagens [14,19,20], nucleic acids [21] and proteins [20]. Moreover, detail about the structure and local environment of these macromolecules can also be elucidated. FTIR spectroscopy requires no pre-analytical chemical modification and is relatively inexpensive, rapid and can operate in an automated fashion. This also allows for tissues to undergo pathological examination after infrared spectroscopy with no danger of the process compromising the tissue. Therefore, FTIR offers an extremely attractive complementary technique in many diagnostic settings.

However, it has become apparent that how the tissue is handled prior to FTIR imaging requires a number of considerations that are relatively unimportant when using conventional microscopy methods [22–24]. These include sample thickness, hydration and the interference caused by external substances such as dyes and culture media[3]. Moreover, infrared spectra of cells and tissue samples are prone to baseline distortions from Mie scattering which is a result of sample morphology [25,26]. FTIR spectroscopy is also limited to a penetrative depth of approximately 10 μm because of detector saturation and thus require either thin sectioning or the measurement performed using attenuated total reflectance (ATR) spectroscopy where the depth of penetration is approximately 3 μm depending on the wavelength, refractive index of the IR window and sample material and the angle of incidence of the IR beam into the crystal.

There is the potential to use this technique to retrospectively analyse archived biological tissues. However, over recent decades it has become apparent that sample collection, processing and preservation have the potential to influence the biochemical spectra of biological tissues (for example Mie scattering effects) [3,27]. Hence, in order to utilize FTIR micro-spectroscopy to accurately assess biological tissue samples, it is imperative to understand how different methods of preparation for analysis impact on the biochemical spectra and thus avoid misleading interpretation of data analysis.

The aim of the present study was to evaluate several different sample preparation techniques for FTIR examination of heart and liver tissue sections (two spectroscopically very different biological tissues) taken from healthy adult rats. The sections were prepared for FTIR analysis by four different methods typically used for histological preparation: A) desiccation dehydration, B) dehydration by ethanol substitution, C) formalin fixation and D) direct measurement of sectioned hydrated tissue.

## Materials and Methods

### Sample preparation for FTIR transflection spectroscopy of dried samples

The collection of organs for this study was harvested from an unrelated project utilising rats and the entire study was specifically approved by the Monash University, ‘School of Biomedical Sciences Animal Ethics Committee A’. The animal care and experimental procedures were carried out in accordance with the Australian ‘Code of Practice for the Care and Use of Animals for Scientific Purposes’. These animals were sacrificed by 100mg/kg pentobarbitone intraperitoneal injection and organs collected immediately afterwards.

At postmortem, liver and heart were immediately excised from adult rats, trimmed of connective tissue and sliced into pieces of approximately 1 mm in thickness. The tissue pieces were immediately transferred into isotonic saline solution. For samples undergoing desiccation, alcohol and formalin fixation, fresh-frozen sectioning was required. To do this, randomly selected pieces were mounted with OCT (optimal cutting temperature) medium onto a cryotome (Leica model CM 3050S, sectioning temperature −25°C) and sectioned. In order to obtain good transflection FTIR spectra of tissue samples, the tissue was sectioned to 5 μm, a level of thickness that allows the infrared light to penetrate. To ensure a high level of homogeneity, sections taken from both liver and heart tissue were taken consecutively and immediately mounted onto Ag/SnO_2_ coated infrared reflective “Mirr-IR” slides (Kevley Technologies). The sample preparation varied according to one of the following three methods:

**A) Desiccator dehydrated tissue.** The fresh tissue pieces were taken from the saline solution, immediately sectioned as described above, mountedon“Mir-IR”slides and placed in a desiccator loaded with silica gel for 48 hours.

**B) Alcohol dehydrated tissue.** The fresh tissue pieces were immersed in absolute alcohol for10 minutes then sectioned as above and allowed to air dry on the “Mirr-IR” slides.

**C) Formalin dehydrated tissue.** The tissue pieces were stored in formalin for 30 days prior to being sectioned and mounted on “Mirr-IR” slides as described above and allowed to air dry.

### Sample preparation for ATR-FTIR spectroscopy of hydrated tissues

**D) Fresh / hydrated tissue.** ATR-FTIR spectra were obtained directly from freshly excised fully hydrated tissue by positioning each tissue piece flush against the sampling ATR crystal.

## Data Acquisition

### FTIR micro-spectroscopy of dried tissue sections

Spectra were collected with an Agilent “Stingray” FTIR micro-spectroscopy system which combines a 7000 series rapid-scan spectrometer, a 600 UMA microscope and a liquid nitrogen cooled mercury-cadmium-telluride 64 x 64 pixel focal plane array (FPA) detector. For each tissue section three spectral maps of 32 x 32 pixels were obtained from different locations within the section. (4 pixel aggregation and 64 co-added scans). Background spectra were collected from a region free of tissue of the FTIR-reflective slide.

### ATR-FTIR spectroscopy of fresh hydrated tissue sections

ATR-FTIR spectra of the hydrated tissue pieces were recorded on a Bruker IFS Equinox FTIR system paired with a Golden Gate single bounce diamond micro-ATR accessory. All FTIR spectra were recorded over the range 4000–800 cm^−1^ at 6 cm^−1^ spectral resolution and with a zero fill factor of 2.

## Data Analysis

The FTIR images acquired of the dried tissue sections were imported into *CytoSpec* (www.cytospec.com) and exported to MATLAB format. The spectra contained within the FTIR images were then corrected over the spectral region 3800–950 cm^−1^ for resonant Mie scatter induced distortion of the baselines using the RMieS-EMSC algorithm (version 3) at the as-acquired 6 cm^−1^ spectral resolution. The correction was completed over 8 iterations modeling for a range of scattering particle radii from 2 to 8 μm, with lower and upper ranges for average refractive indices set at 1.1 and 1.5 respectively and using 10 equidistant values for constructing the Mie scattering curve matrix compressed to 7 orthogonal principal components.

For the dried tissue sections average spectra were calculated for each tissue section. There was no significant variation between the average spectra from the individual tissue sections of similar tissue type that had received the same treatments, and so, a group average spectrum for each of the preparation methods for each of the two tissue types was then generated. Average second derivative spectra were calculated from the group average spectra using the Savitsky-Golay algorithm (9 smoothing points) in order to enable better elucidation of the overlapping band features within the spectra.

Average ATR-FTIR spectra for each tissue type were calculated from the spectra obtained from the fresh hydrated tissue pieces. Second derivative spectra were computed using the Savitsky-Golay algorithm (9 smoothing points). No other preprocessing steps were performed on the ATR-FTIR spectra.

## Results and Discussion

**Fig 1:**
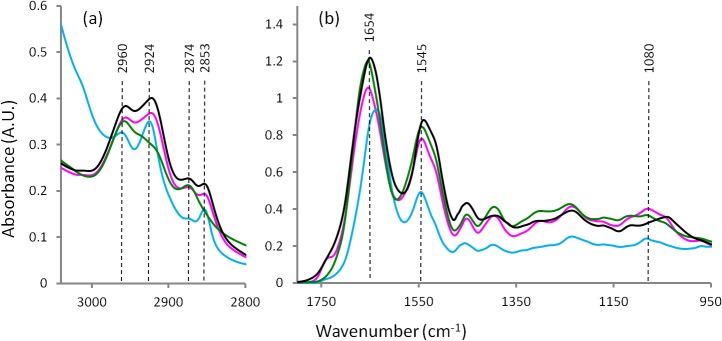
Average FTIR spectra from heart tissue: hydrated tissue ATR spectrum (blue), formalin fixed transmission spectrum (black), desiccator dried transmission spectrum (pink) and ethanol dehydrated transmission spectrum (green).

**Fig 2:**
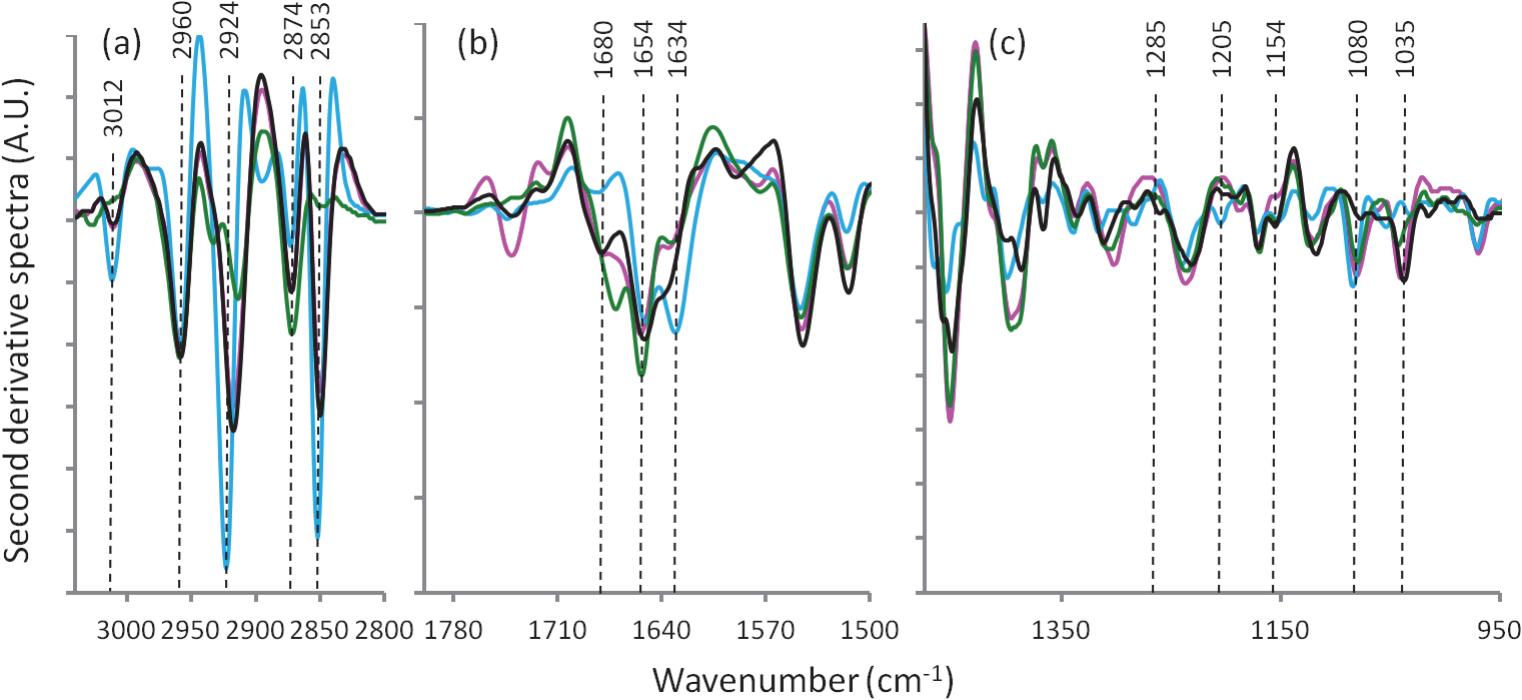
Second derivative spectra calculated from Fig. 1 for heart tissue: hydrated tissue ATR spectrum (blue), formalin fixed transmission spectrum (black), desiccator dried transmission spectrum (pink) and ethanol dehydrated transmission spectrum (green).

**Fig 3:**
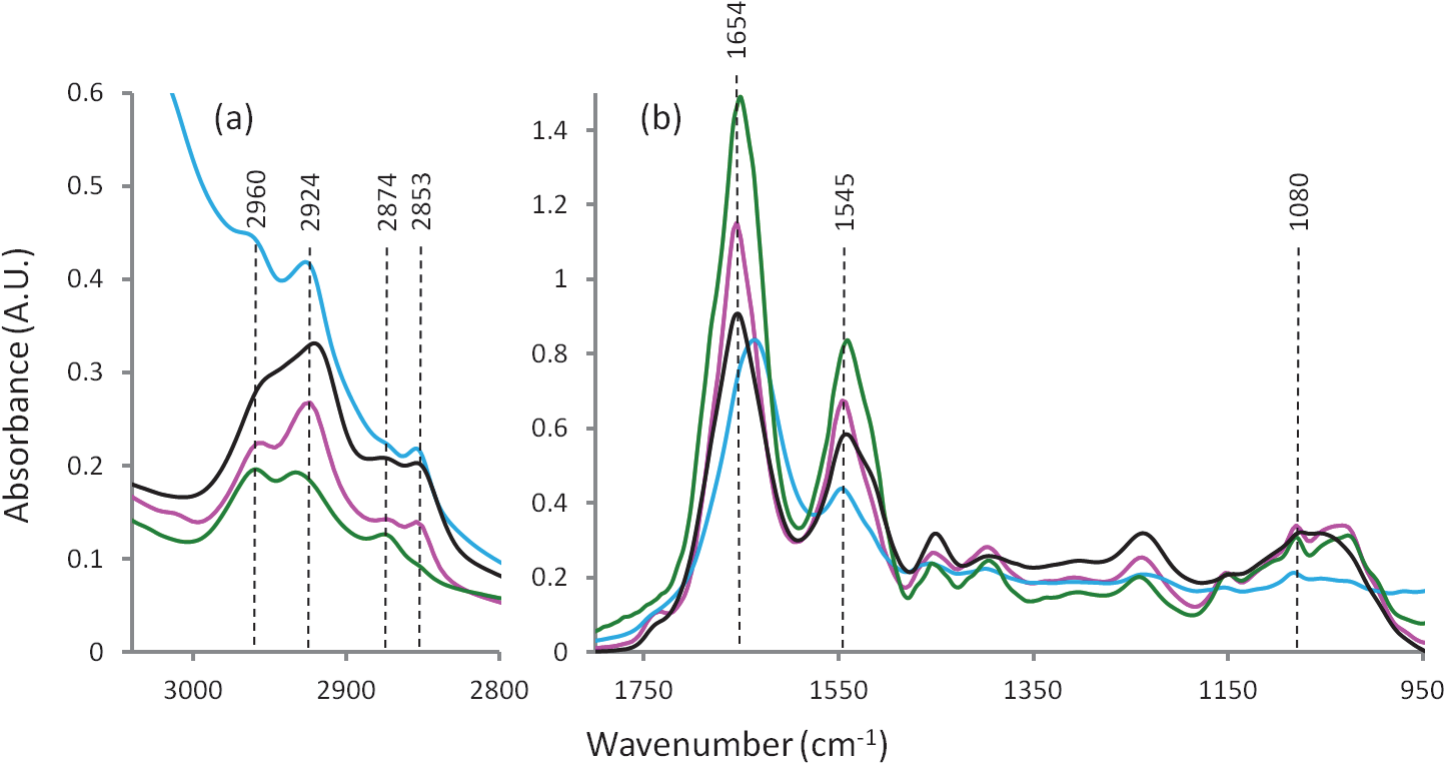
Average FTIR spectra from liver tissue: hydrated tissue ATR spectrum (blue), formalin fixed transmission spectrum (black), desiccator dried transmission spectrum (pink) and ethanol dehydrated transmission spectrum (green).

**Fig 4:**
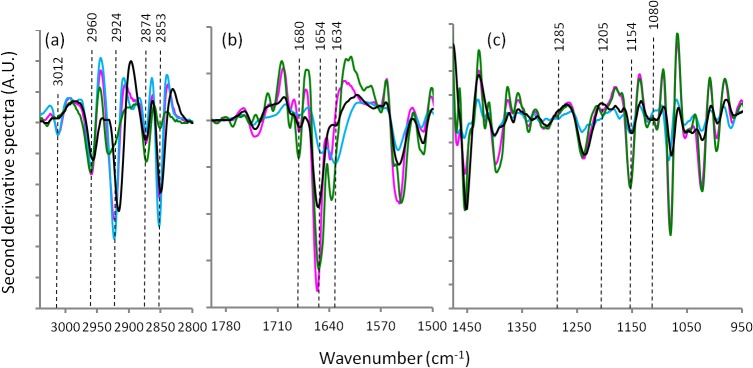
Second derivative spectra from Fig. 3 for liver tissue: hydrated tissue ATR spectrum (blue), formalin fixed transmission spectrum (black), desiccator dried transmission spectrum (pink) and ethanol dehydrated transmission spectrum (green).

Fig. 1 represents the average FTIR spectra for the heart tissues prepared by the four different techniques. Fig. 2 shows the second derivative spectra of the heart tissues spectra in Fig. 1. Figs. 3 and 4 present the average FTIR spectra and the corresponding second derivative spectra from the liver tissues, respectively. From the spectra obtained for the four different preparation methods it was apparent, especially in the second derivative spectra, that there were many bands exhibiting significant shifts of the absorption band positions compared to the fresh / hydrated tissue. Tables 1 and 2 summarize the peak wavenumber values and their assignments for the important features for the heart and liver tissues, respectively. By considering, in turn, the groups of characteristic bands associated with the key macromolecules the relative effectiveness of the three methods for dehydrating the samples while preserving the biochemistry can be determined. Here the average ATR-FTIR spectrum of the fresh hydrated tissues (Figs. 1 and 3 blue trace) can be considered as the gold standard for preservation of biochemistry. However, it is important to note that because of the strong, broad absorptions of the water located at 1625 and 3200 cm^−1^, there is some degree of overlap, particularly of the amide I, and a loss of intensity in many absorption bands. Because of this, accurate measurement of peak wavenumber values and intensities are not always possible. The hydrated tissue spectra also exhibited marked differences in the relative strengths of various bands when compared with the dried tissue spectra. It has recently been reported that the extinction coefficients for the bands associated with the nucleic acids are significantly changed when these molecules are taken from hydrated to dehydrated states [11]. It is not unreasonable to assume that the same could be true for other biological macromolecules.

**Table 1:** Band assignments for the heart tissue spectra.

Desiccated (pink) (cm^−1^)	Ethanol (green) (cm^−1^)	Formalin (black) (cm^−1^)	Fresh/Wet (blue) (cm^−1^)	Assignment
3012	-	3012	3012	C-H ring, CH_2_ aromatic, ν = C-H lipids
2960	2960	2960	2960	asymmetric CH_3_
2873	2873	2873	2873	symmetric CH_3_ stretch
2851	2851	2851	2853	symmetric CH_2_ stretch
1681	1671	1681	1683	amide I—antiparallel β sheet / random coils
1654	1654	1652	1652	amide I—α-helix
1634	1634	1638	-	amide I—β-sheet
1546	1546	1546	1546	amide II
1391/1397	1391/1397	1387/1401	1401/1420	symmetric methyl (CH_3_) bending proteins, nucleic acids and lipids
1340	1340	1336	1340	collagen
1303	1307	1312	1317	amide III / collagen
-	1285	1285	1285	collagen
-	1200	1200	1205	collagen
1171	1170	1171	1172	collagen
1155	1155	1155	1151	glycogen
1081	1081	1077	1084	ν-PO_2_-
1042	1043	1038	1054/1030	glycogen/ nucleic acids (ν-PO_2_-)
971	971	965	971	ν-PO_4_ of nucleic acids and proteins

**Table 2:** Band assignments for the liver tissue spectra.

Desiccated (pink) (cm^−1^)	Ethanol (green) (cm^−1^)	Formalin (black) (cm^−1^)	Wet/Fresh (blue) (cm^−1^)	Assignment
3012	-	-	3012/2985	C-H ring, CH_2_ aromatic, ν = C-H lipids
2960	2961	2957	2960	asymmetric CH_3_
2924	2932/2924	2938/2917	2924	cholesterol, phospholipids / asymmetric CH_2_
2874	2874	2874	2874	symmetric CH_3_ stretch
1682	1682	1680	1682	amide I—antiparallel β sheet / random coils
1657	1655	1655	1651	amide I—α-helix
1639	1638	1631	1633	amide I—β-sheet
1594	1594	-	-	C = N, NH_2_ adenine
1571	1571	1581/1569	1570 (weak)	C = N adenine
1547	1553/1543	1547	1547	amide II
1513	1513	1513	1515	amide II / carotenoid pigment
1454	1454	1452	1454	asymmetric CH_3_
1397	1397	1400/1384	1400	symmetric methyl (CH_3_) bending proteins, nucleic acids and lipids
1338	1338	1332	1340	collagen
1311	1312/1304	1305	1317	amide III / collagen
1285	1285	1285	1283	collagen
1238	1240	1236	1240	stretching PO_2_ asymmetric nucleic acid / amide III
1204	1204	-	1204	collagen
1172	1172	1170	1172	collagen
1154	1154	1150	1154	glycogen
1122	1123	1118	1120	symmetric phosphodiester RNA / C-O carbohydrates
1080	1080	1078	1084	ν-PO_2_-
971	971	965	971	ν-PO_4_ of nucleic acids and proteins

### Protein bands

The spectral region 1800–1500 cm^−1^ (Fig. 2(B) and Fig. 4(B)) corresponds primarily to absorptions attributable to proteins. The principle protein marker band is the amide I absorption centered around 1654 cm^−1^. The amide I band position and shape is sensitive to the secondary structures of proteins [28] but is also particularly susceptible to distortions from light scattering effects and hence, it is essential that such distortions are removed to avoid misinterpretation of the protein secondary structure information [26]. In the second derivative spectra this band was resolved into a set of 3 amide I peaks at ∼1680, 1654 and 1634 cm^−1^. In the hydrated tissue sample’s ATR-FTIR spectrum the1634 cm^−1^band appeared strong and was shifted to ∼1631 cm^−1^: this is due to the presence of the underlying OH bending mode of water at ∼1633 cm^−1^. In comparing the amide I band in the second derivative spectra of the heart and liver tissues, marked differences are apparent in the relative strengths of the 3 underlying band features. This is due to the different overall protein composition of the two tissue types. For both tissue types and for all sample preparation methods the central and strongest peak (∼1654 cm^−1^) is associated with both α-helical secondary structure and random disordered structure [29,30]. The position and intensity of this peak was largely unchanged except for in the formalin fixed liver tissue where the 1654 cm^−1^ absorption appeared weaker in intensity. An amide I absorption associated with β-sheet protein secondary structure was apparent in the ethanol fixed and desiccated heart tissues at ∼1634 cm^−1^but was blue shifted to 1638 cm^−1^ in the formalin fixed heart tissue samples. Conversely, this same β-sheet band was observed at ∼1638 cm^−1^ in the ethanol fixed and desiccated liver tissues but was red shifted to 1631 cm^−1^ in the formalin fixed liver tissues. The highest wavenumber value component peak of the amide I group was the absorption at 1681 cm^−1^ associated with β-turn secondary structure. For both tissue types this band changed only slightly between samples dried by desiccation or fixed with formalin but for the samples prepared using water substitution by ethanol there was a significant increase in the intensity of this peak for both tissue types and for the heart tissue a shift to a lower frequency (1671 cm^−1^) was also observed.

Considering these observed changes in the underlying features of the amide I peak together, and considering the varied mixture of protein content present in these tissue samples, it is not possible from this work alone to unambiguously determine what changes in protein secondary structure or protein folding or unfolding events may have occurred as a result of the various applied sample preparation treatments. However, changes in the profile and intensity of the amide I band were clearly observed for both the formalin fixed and ethanol substitution dried samples compared with the desiccator dried samples and the ATR-FTIR spectra obtained from hydrated samples. The changes in the amide I band profile reflect significant differences in hydrogen-bonding strengths and differences in transition dipole coupling as these are the factors that give the amide I band its sensitivity to protein secondary conformational changes. Both the formalin fixation and the ethanol dehydration methods altered the amide I band profile and thus affected its usefulness for reliable determination of percentages of secondary structures present in the tissues. These methods are indicating that they do not reflect the true living native hydrated state of the proteins. A significant reduction in the intensity of the amide I peak was observed with formalin fixation. This has previously been reported by Faolain and colleagues and attributed to a loss of secondary amide upon formation of tertiary amides through the cross linking action of formalin [27]. In the same work, a shift of the amide I peak following tissue fixation with formalin was also reported as was observed in this work.

Mie scatter induced red shifts of these bands may have been a difficulty that was encountered in earlier studies. At that time the spectrum distorting influence of scattering phenomena was not understood fully and algorithmic corrections were not available. In the current work, scattering effects have been removed prior to the analysis and interpretation of the spectra. A shift of the amide I and amide II bands to lower wavenumber values after ethanol fixation for preservation of bone that was previously reported by Pleshko et al [31] and cited in later works [32] but it was not observed in our work. Pleshko’s assignment to ethanol induced hydrogen-bonding changes and/or protein secondary conformational changes is therefore not of general applicability to all protein mixtures and may be a mistaken attribution of Mie scatter induced spectral changes. In addition, they reported that formalin fixation did not alter the amide I band profile, contrary to our observations.

### Lipid bands

The spectral region 3100–2800 cm^−1^ (Fig. 2(A) and Fig. 4(A)) corresponds to lipids.The 3012 cm^−1^ band assigned to olefins or unsaturated fatty acids [33,34] were observed in the FTIR-ATR spectra obtained from both types of hydrated tissues. In both tissue types this band was absent following ethanol dehydration. In the heart tissue this band was detected following sample preparation by both formalin fixation and by desiccator drying. But in the liver tissue this band was absent following formalin fixation. Similarly, the bands at 2924 and 2853 cm^−1^ assigned to ν_as_CH_2_ and ν_s_CH_2_ of lipids, respectively, were greatly decreased in intensity or absent for both tissue types when ethanol was used in the sample preparation. Presumably, this indicates complete extraction of lipids as a result of the ethanol water substitution treatment. It was also observed that these bands were slightly red shifted (∼2 cm^−1^) when formalin fixation had been used. The two bands at 2960 and 2874 cm^−1^ were relatively unchanged in both tissue types and for all sample preparation methods. These ν_as_CH_3_ and ν_s_CH_3_ absorptions are often assigned to lipids and also methyl groups contained in nucleic acids and proteins. Given that the CH_2_ bands were absent in the ethanol treated samples due to lipid extraction, these bands must be predominantly due to methyl groups contained in either proteins or nucleic acids or both and are therefore relatively insensitive to lipid content, at least in these samples.

### Mixed contribution region—carbohydrates, proteins, nucleic acids

The spectral region 1475–950 cm^−1^ (Fig. 2(C) and Fig. 4(C)) corresponds to the bands from several macromolecules: carbohydrates, proteins and nucleic acids. The second derivative spectra in the region 1390–1475 cm^−1^ exhibited a complex mixture of band features for both tissue types. These absorptions are predominately due to bending modes of CH_2_ and CH_3_ groups. For both tissue types the desiccated and the ethanol treated samples demonstrate absorptions that were remarkably similar suggesting that this region is also not particularly sensitive to lipid content. Comparing the hydrated ATR spectra and the formalin fixed tissue spectra with the other two dried tissue preparations, significant wave number shifts and splitting of the CH_2_ and CH_3_ band features were observed (Table 1 and Table 2). In the case of the formalin fixed tissues, this can be attributed to the cross-linking action of the fixative on proteins. The spectra of the formalin fixed tissues also exhibited band shifts for many of the peaks in the spectral region from approximately 1330–1150 cm^−1^ which can be associated with amide III modes and in particular with the spectrum of collagen.

In the heart tissue spectra two bands characteristic of collagen at 1205 and 1285 cm^−1^ were observable in the wet tissue spectra but only weakly detected in the formalin fixed and ethanol dehydrated tissue spectra. These collagen marker bands were apparently absent from the desiccated sample spectra. Bands in the region 1160–950 cm^−1^ are largely due to stretching vibrations of CO groups in carbohydrates although CO in the ribose backbone of nucleic acids and the ν_s_PO_2_^-^ (symmetric phosphate stretching) modes also contribute absorbance at around 1080 cm^−1^. In the desiccated and ethanol treated liver tissue samples the triplet of bands due to glycogen (1154, 1080, 1035, 1022 cm^−1^) appeared strong in the spectra but were weak in the wet spectra and altered to a broad band feature in the formalin fixed tissues. Establishment of glycogen levels is important for the diagnosis of many diseases [35,36]. Clearly the spectra indicate that formalin fixation has some effect on glycogen chemistry in tissues. In this regard, there are conflicting reports in the literature in relation to the effects of formalin on tissue glycogen. Formalin has been used as a method for assay extraction of glycogen from tissues [37], whereas others have reported that formalin is effective for localizing and preserving glycogen content in tissues [38]. Our findings support the latter; there was no evidence that formalin removes glycogen from either the heart or liver tissue.

## Conclusions

To our knowledge, this is the first time in the FTIR micro-spectroscopic examination of mammalian tissues, that the spectroscopically detectable variations in sample chemistry arising from different preparation techniques have been described. The choice of sample preparation and fixation should be made carefully when commencing new studies on these tissue types to ensure that the macromolecular content of interest is not unduly perturbed by the sample preparation process. Prospectively, the best approach is to use freshly excised tissues for immediate analysis. This is not to say however that studies based on the extremely valuable and pre-existing collections of archived tissue should not be conducted. Rather, care must be taken to ensure that the biochemical alterations which have occurred in preparing these tissues for archiving are fully understood and considered.
